# Locked Nucleic Acid Gapmers and Conjugates Potently Silence *ADAM33*, an Asthma-Associated Metalloprotease with Nuclear-Localized mRNA

**DOI:** 10.1016/j.omtn.2017.06.012

**Published:** 2017-06-21

**Authors:** Hannah M. Pendergraff, Pranathi Meda Krishnamurthy, Alexandre J. Debacker, Michael P. Moazami, Vivek K. Sharma, Liisa Niitsoo, Yong Yu, Yen Nee Tan, Hans Michael Haitchi, Jonathan K. Watts

**Affiliations:** 1 Department of Chemistry, University of Southampton, Southampton SO17 1BJ, UK; 2 Institute for Life Sciences, University of Southampton, Southampton SO17 1BJ, UK; 3 RNA Therapeutics Institute, UMass Medical School, Worcester, MA 01605, USA; 4 Department of Biochemistry and Molecular Pharmacology, UMass Medical School, Worcester, MA 01605, USA; 5 Institute of Materials Research and Engineering, A*STAR, Singapore 138634, Singapore; 6 Clinical and Experimental Sciences, Faculty of Medicine, University of Southampton, Southampton SO16 6YD, UK; 7 NIHR Southampton Respiratory Biomedical Research Unit at University Hospital Southampton NHS Foundation Trust, Southampton, Southampton SO16 6YD, UK

**Keywords:** ADAM33, gene silencing, antisense, siRNAs, nuclear RNA

## Abstract

Two mechanisms dominate the clinical pipeline for oligonucleotide-based gene silencing, namely, the antisense approach that recruits RNase H to cleave target RNA and the RNAi approach that recruits the RISC complex to cleave target RNA. Multiple chemical designs can be used to elicit each pathway. We compare the silencing of the asthma susceptibility gene *ADAM33* in MRC-5 lung fibroblasts using four classes of gene silencing agents, two that use each mechanism: traditional duplex small interfering RNAs (siRNAs), single-stranded small interfering RNAs (ss-siRNAs), locked nucleic acid (LNA) gapmer antisense oligonucleotides (ASOs), and novel hexadecyloxypropyl conjugates of the ASOs. Of these designs, the gapmer ASOs emerged as lead compounds for silencing *ADAM33* expression: several gapmer ASOs showed subnanomolar potency when transfected with cationic lipid and low micromolar potency with no toxicity when delivered gymnotically. The preferential susceptibility of *ADAM33* mRNA to silencing by RNase H may be related to the high degree of nuclear retention observed for this mRNA. Dynamic light scattering data showed that the hexadecyloxypropyl ASO conjugates self-assemble into clusters. These conjugates showed reduced potency relative to unconjugated ASOs unless the lipophilic tail was conjugated to the ASO using a biocleavable linkage. Finally, based on the lead ASOs from (human) MRC-5 cells, we developed a series of homologous ASOs targeting mouse *Adam33* with excellent activity. Our work confirms that ASO-based gene silencing of *ADAM33* is a useful tool for asthma research and therapy.

## Introduction

Asthma is a chronic respiratory disease involving airway inflammation and structural changes in the form of remodeling of the conducting airways.^1^, ^2^ It causes more than 345,000 deaths annually and affects more than 340 million people worldwide.^3^ *ADAM33* is the first asthma susceptibility gene to be identified by positional cloning.^4^ Single-nucleotide polymorphisms in *ADAM33* have been linked to asthma and bronchial hyperresponsiveness.^4^, ^5^ *ADAM33* encodes a membrane-anchored metalloprotease, but a soluble metalloprotease-containing form of ADAM33 (sADAM33) is increased in bronchoalveolar lavage fluid of asthmatic patients.^6^, ^7^ Furthermore, in human embryonic lung explant culture, treatment with sADAM33 induces angiogenesis^8^ and myogenesis,^7^ pathological features of airway remodeling in asthma. Expression of human sADAM33 in transgenic mice causes pathological airway remodeling and makes airways more susceptible to allergen-induced inflammatory responses.^7^ Promisingly, when induction of human sADAM33 is arrested, this leads to reversal of the remodeling and reduced sensitivity to inflammatory responses.^7^ Furthermore, in *Adam33*-null mice challenged with allergen, both airway remodeling and inflammation are suppressed. Therefore, inhibition of *ADAM33* represents an attractive disease-modifying therapeutic strategy for treating the root cause of asthma in many patients.

**Figure 1:**
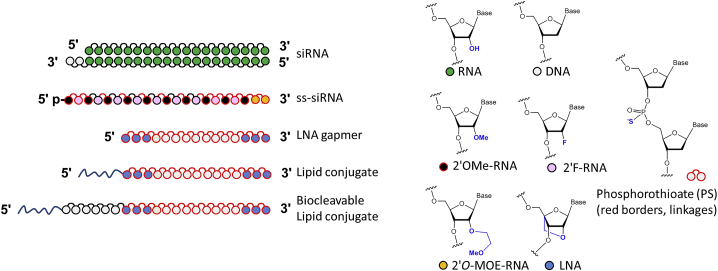
Types of Gene Silencing Oligonucleotides and Chemical Modifications Used in This Study Types of gene silencing oligonucleotides (left) and chemical modifications (right) used in this study. p, phosphate.

It is thus crucial to develop therapeutic agents capable of silencing of *ADAM33* expression. Because small molecule metalloprotease inhibitors have shown a poor specificity profile,^9^ chemically optimized oligonucleotide-based inhibitors could serve as an excellent alternative.^10^, ^11^, ^12^ In this study, we compared four types of oligonucleotide-based gene silencing agents for inhibition of *ADAM33*/*Adam33* (Figure 1): (1) duplex small interfering RNAs (siRNAs), (2) chemically modified single-stranded small interfering RNAs (ss-siRNAs) that operate by the RNA-induced silencing complex (RISC) pathway,^13^, ^14^ (3) locked nucleic acid (LNA) gapmer ASOs operating via the RNase H mechanism,^15^, ^16^, ^17^ and (4) novel biocleavable lipid conjugates of the potent ASOs (including biocleavable conjugates). The LNA gapmers, delivered using cationic lipid or by gymnosis,^18^ provided significantly increased potency for silencing *ADAM33* relative to the siRNAs or ss-siRNAs.

## Results

### *ADAM33* Silencing by siRNAs and ss-siRNAs

**Figure 2:**
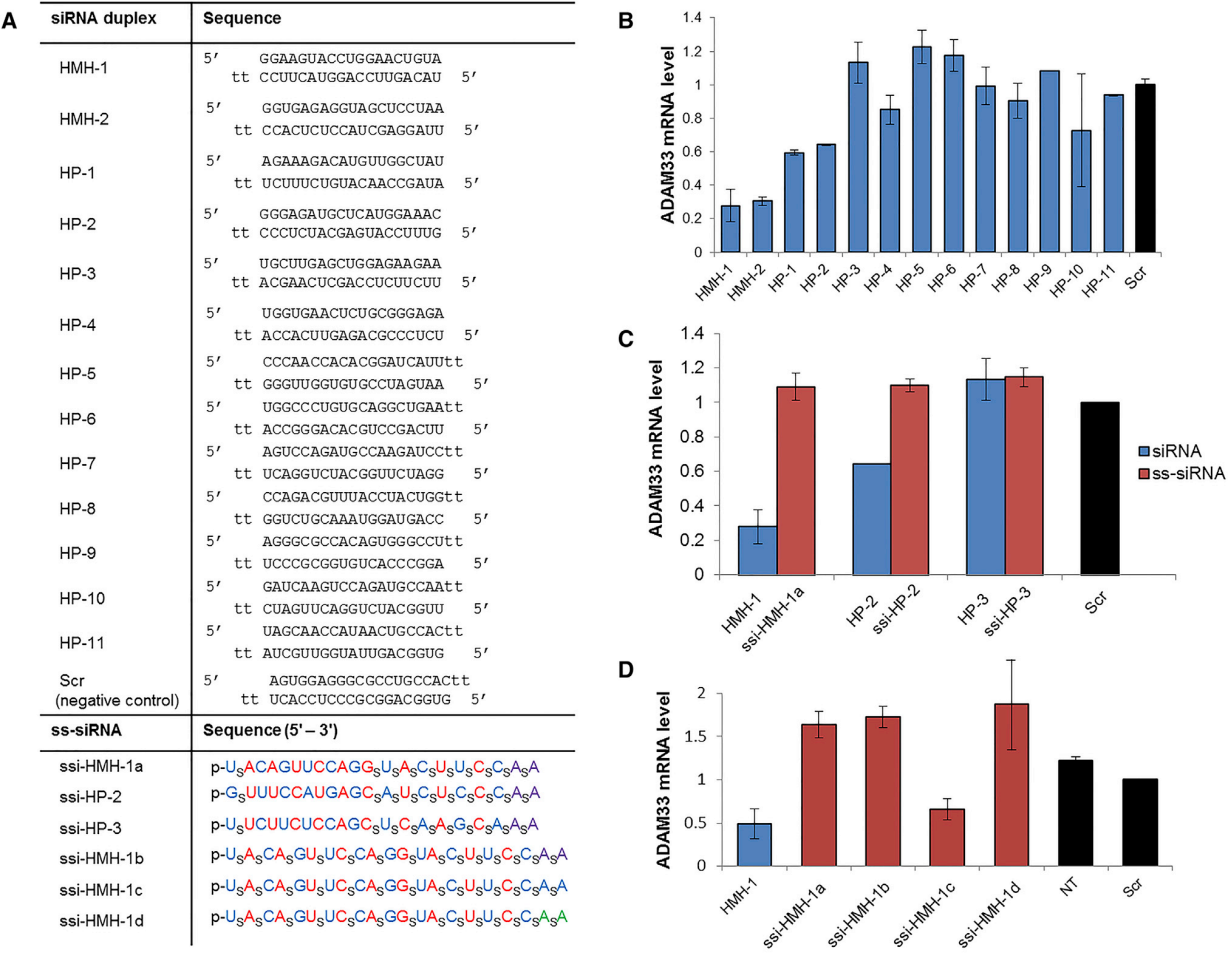
*ADAM33* Gene Expression Can Be Inhibited by siRNA and ss-siRNA Oligonucleotides, but the Maximal Extent of Inhibition Is Modest (A) Oligonucleotide sequences: tt represents a 3′ terminal overhang of two deoxy thymidines; duplex siRNAs are otherwise unmodified RNA. The ss-siRNA sequence modifications are subscript s, phosphorothioate linkage; red, 2′F-RNA; blue, 2′OMe-RNA; purple, 2′-*O*-MOE-RNA; green, LNA; p, 5′-phosphate. For siRNA duplexes, passenger strands are listed on top and guide strands underneath. (B) qRT-PCR results from a screen of siRNA duplexes showing that few siRNA sequences were active against this target. (C) qRT-PCR results showing the gene silencing efficacy of ss-siRNA analogs of the three most potent duplex siRNAs, in comparison with the parent duplexes. (D) qRT-PCR results comparing potencies of different chemical modification schemes on ss-siRNA activity. Error bars represent the SD of biological replicates. All oligonucleotides were transfected at 50 nM into MRC-5 lung fibroblasts using Lipofectamine RNAiMAX. All results are normalized to a scrambled siRNA duplex control.

We designed and synthesized a panel of 13 duplex siRNAs targeting different regions of the *ADAM33* transcript (Figure 2A). We then tested the efficacy of these siRNAs in MRC-5 human lung fibroblast cells, transfecting the siRNAs with a lipid transfection reagent and measuring silencing by qPCR. We found that most of the sequences were inactive, while the most active duplexes were able to attain about 70% silencing (Figure 2B).

Single-stranded oligonucleotides can be recognized by the RNAi machinery and serve as guide strands, if the oligomer is stabilized to resist nuclease cleavage and contains a 5′-phosphate or phosphonate.^13^, ^14^, ^19^, ^20^ To explore whether changing the biophysical properties of the RNAi trigger could improve activity, we synthesized and tested ss-siRNA analogs of three siRNA sequences: highly active sequence HMH-1, moderately active sequence HP-2, and inactive sequence HP-3. The ss-siRNA analogs showed reduced efficacy (HMH-1 and HP-2) or maintained inactivity (HP-3) (Figure 2C). For the most active siRNA sequence, HMH-1, we carried out a chemical optimization of the ss-siRNAs and found one analog with activity that was comparable, but not superior, to the parent duplex RNA (Figure 2D). Only the ss-siRNA with 2′-*O*-methyl-RNA (2′OMe-RNA) modifications at the 3′ terminus was able to approach the potency of the duplex siRNA (ssi-HMH-1c) (Figure 2D). This result was followed up in the context of other sequences and targets, and this exploration of the medicinal chemistry of ss-siRNAs was published.^21^ Overall, neither siRNAs nor ss-siRNAs were able to surpass 70% silencing of the *ADAM33* transcript under any of the conditions we tested.

To ensure that the relatively limited silencing we observed was not an artifact of either of the preceding designs, we synthesized and tested a third option for siRNA design—i.e., fully chemically modified duplex siRNAs. We applied an alternating pattern of 2′OMe-RNA and 2′-fluoro-RNA (2′F-RNA) to the HMH-1 sequence, using a 5′-phosphorylated 21-mer guide strand and a 15-nt sense strand (Figure S1). This and similar designs have been widely used and show excellent results both in vitro and in vivo.^22^, ^23^, ^24^, ^25^, ^26^ The fully modified duplex was transfected into MRC-5 cells, and its efficacy was comparable to the parent unmodified siRNA duplex (Figure S1).

### LNA Gapmers Show High Potency when Transfected with a Cationic Lipid

**Figure 3:**
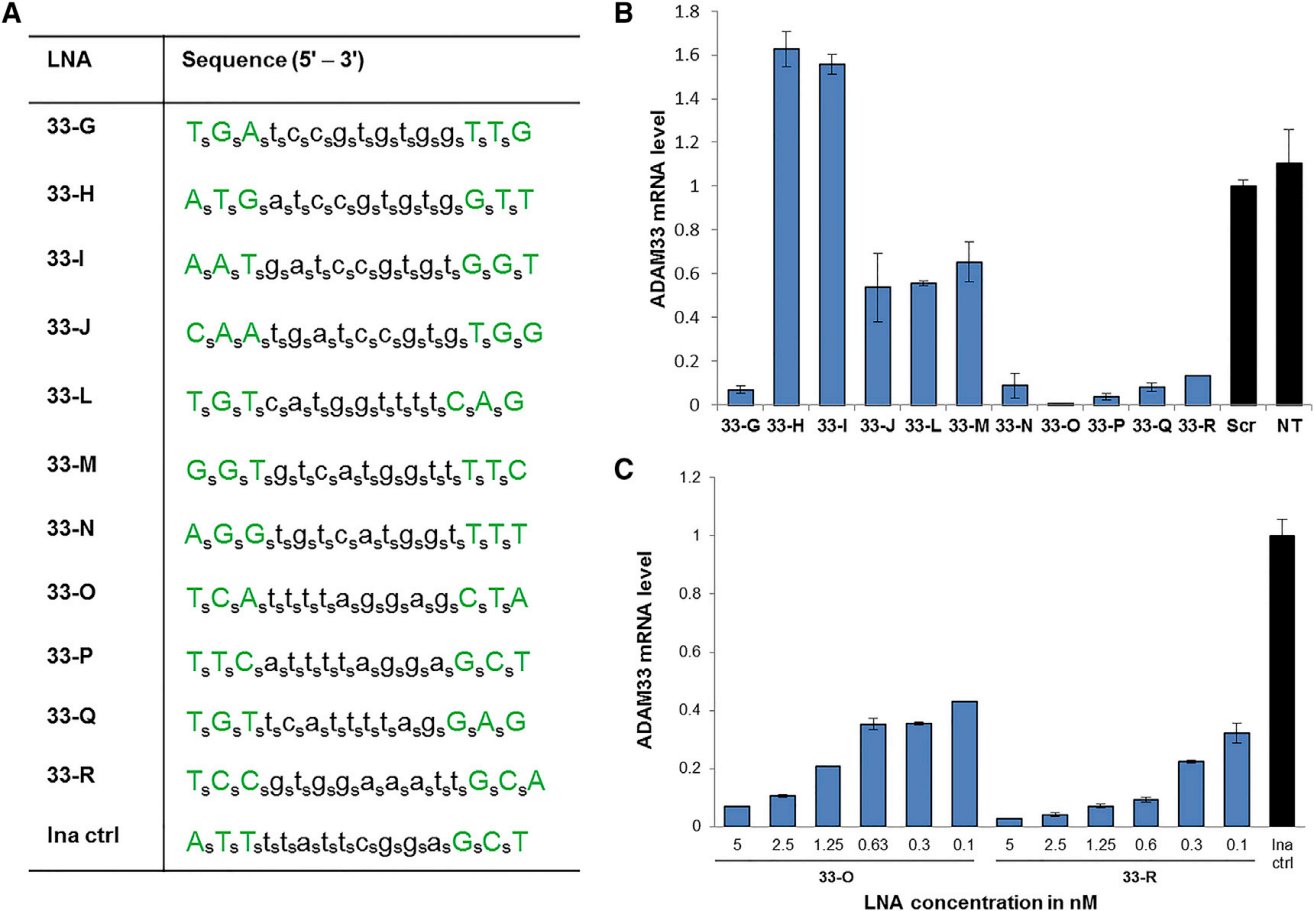
LNA Gapmers Are Highly Potent when Transfected with a Cationic Lipid (A) LNA gapmer sequence modifications are subscript s, phosphorothioate linkage; green uppercase, LNA; lowercase, DNA. (B) qRT-PCR results showing gene silencing by LNA gapmers at 50 nM. All results are normalized to a scrambled siRNA duplex control. Error bars represent the SD of biological replicates. (C) qRT-PCR results showing dose-response analysis of 33-O and 33-R. Results are normalized to a scrambled LNA gapmer control. Error bars for the dose responses represent the SD of technical replicates.

Another major class of single-stranded gene silencing oligonucleotides consists of gapmer ASOs.^27^, ^28^, ^29^ Gapmer ASOs operate through a mechanism different from the preceding siRNAs and ss-siRNAs: they recruit RNase H to cleave target mRNAs.^30^, ^31^, ^32^ The “wings” of gapmers consist of sugar-modified nucleotides such as 2′-*O*-methoxyethyl-RNA (2′-*O*-MOE-RNA)^33^ or LNA,^34^, ^35^, ^36^ which increase binding affinity and nuclease stability, while the central “gap” is phosphorothioate (PS) DNA, which elicits RNase H cleavage of the target. We designed and synthesized 12 PS LNA gapmers, using a 3-9-3 pattern of sugars and targeting various regions of *ADAM33* mRNA (Figure 3A). We initially tested the LNA gapmers at a 50 nM concentration in MRC-5 fibroblasts, using Lipofectamine RNAiMAX as a transfection agent. LNAs 33-G, 33-N, 33-O, 33-P, 33-Q, and 33-R all achieved >80% silencing of *ADAM33* when normalized to a scrambled siRNA duplex control (Figure 3B). This very high hit rate and maximal efficacy contrast sharply with the low hit rate and maximal efficacy observed when using RISC-dependent oligonucleotides (siRNAs and ss-siRNAs) for this target. Furthermore, a dose-response analysis shows potent, dose-dependent *ADAM33* inhibition for 33-O and 33-R to concentrations < 0.16 nM (Figure 3C).

### Gymnotic Delivery of LNA Gapmers

Although the LNA gapmers were highly potent in cultured cells, transfection efficiency in vitro does not always correlate well with in vivo studies. Ex vivo lung tissue and in vivo lung experiments also require oligonucleotides with the ability to exert potent silencing without the aid of toxic transfection agents. Naked LNA or 2′-fluoroarabinonucleic acid (2′F-ANA) ASOs can be taken up by most types of dividing cells in culture in a process termed gymnosis.^18^, ^37^, ^38^ Gymnotic delivery does not require serum additives or transfection reagents but is a slower process compared to lipofection. It tends to show low toxicity and shows an improved correlation between in vitro and in vivo results.^18^

The gymnotic approach was adopted to test (1) whether the LNA gapmers could enter the MRC-5 cells without the use of transfection agents, (2) how the potency of the LNA gapmers delivered via gymnotic delivery compared to the transfections using a cationic lipid, and (3) whether the gymnotically delivered LNA gapmers were toxic to cultured cells.

**Figure 4:**
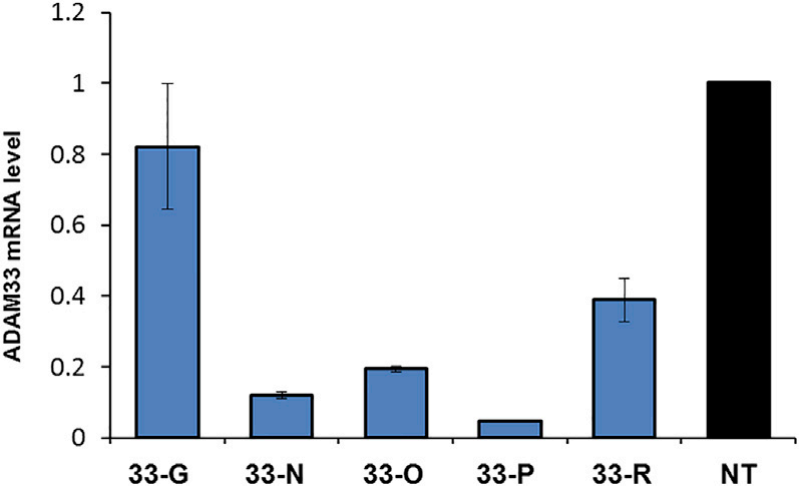
Gymnotically Delivered Gapmers Are Potent Inhibitors of *ADAM33* Gene silencing measured by qRT-PCR after gymnotic delivery. Oligonucleotides are delivered to MRC-5 cells at a 3 μM dose and normalized to a non-treated control (NT). Error bars represent the SD of biological replicates.

A time course experiment was performed, gymnotically delivering 33-O into MRC-5 cells at a 1 μM dose to determine the optimal day to harvest the cells after treatment, between day 4 and day 9. Our results indicate that day 7 post-treatment was an appropriate time to harvest cells (Figure S2). We then selected LNA gapmers 33-G, 33-N, 33-O, 33-P, and 33-R for further testing. Oligomers 33-N, 33-O, and 33-P achieved >80% reduction of *ADAM33* transcript levels, while 33-R showed 60% *ADAM33* silencing at a 3 μM dose (Figure 4).

The LNA gapmers are able to efficiently silence *ADAM33* expression without the aid of transfection agents. Although the potency of the gymnotically delivered oligonucleotides was not as high as the cationic lipid-mediated LNA delivery, the gymnotically delivered LNAs showed no toxicity to cultured cells (based on phenotype and cell confluence).

### Hexadecyloxypropyl Conjugates for Cellular Uptake

One of the major limitations to oligonucleotides as therapeutic agents is their relatively poor uptake into most cells.^39^ One promising solution is the covalent attachment of a ligand that allows recognition by cell-surface receptors.^40^, ^41^, ^42^ We sought to improve uptake by attaching a lipid moiety based on 1-*O*-hexa-decyloxy-1,3-propanediol, which has been previously shown to increase small molecule uptake by MRC-5 fibroblast cells^43^ and improve the oral bioavailability of nucleoside drugs.^44^

**Figure 5:**
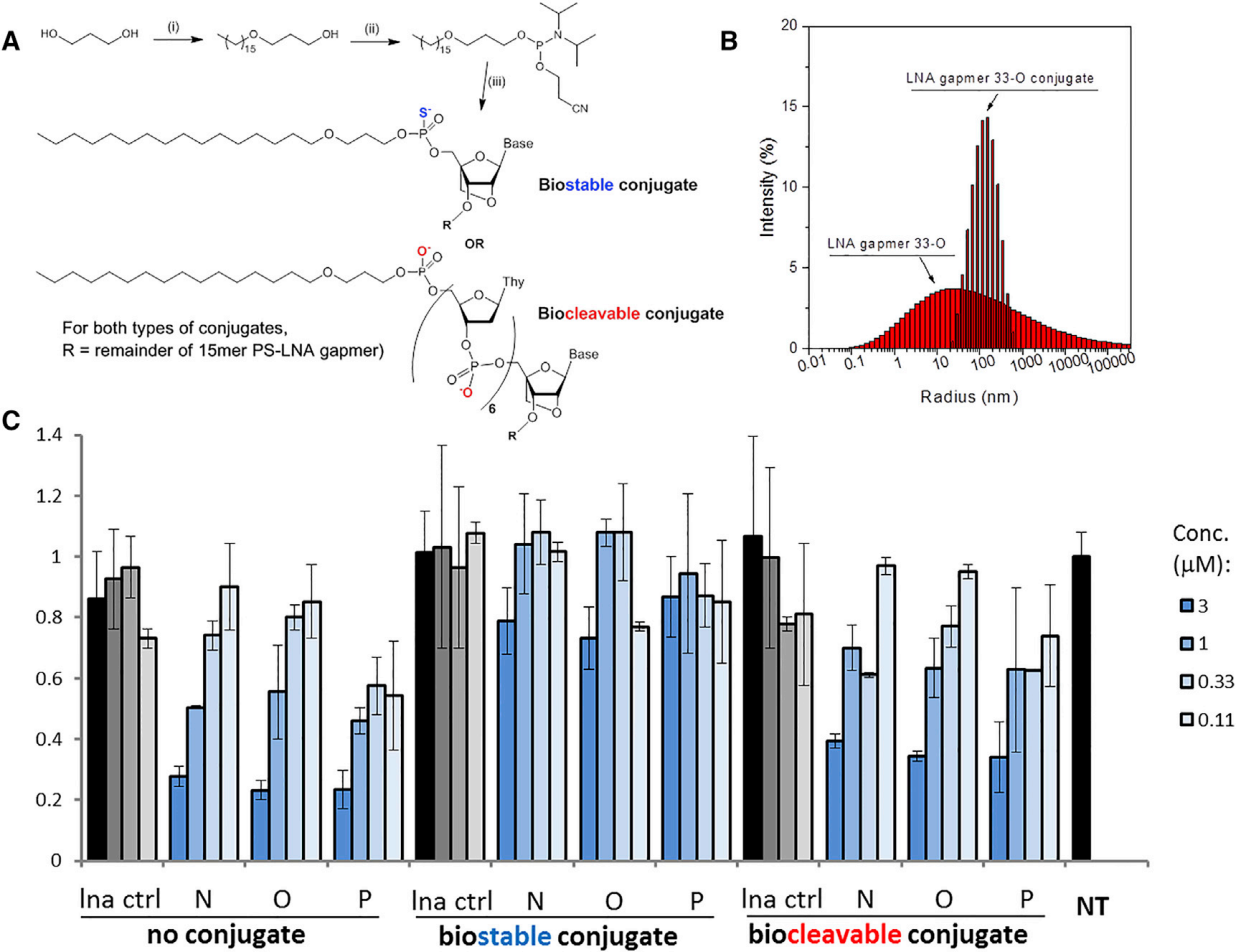
Lipid-Conjugated LNA Gapmers Show Reduced Potency Unless the Lipid Is Joined to the ASO via a Biocleavable Linkage (A) Synthesis and structure of hexadecyloxypropyl LNA conjugates. Reagents and conditions: (i) NaH, CH_3_(CH_2_)_15_Br, cat. KI; (ii) ^i^Pr_2_NP(O(CH_2_)_2_CN)Cl, DIPEA, CH_2_Cl_2_; (iii) standard oligonucleotide synthesis conditions. (B) Dynamic light scattering shows that addition of a lipid tail causes self-assembly of ASOs into larger structures (comparing 33-O, red bars, with its biostable conjugate, black bars). (C) qRT-PCR results showing dose-response analysis of A33-N, A33-O, and A33-P and both types of lipid conjugates after gymnotic delivery to MRC-5 cells at concentrations from 3 to 0.11 μM. Results are normalized to a non-treated control sample (NT). Error bars represent the SD of biological replicates.

A 1-*O*-hexadecylpropanediol phosphoramidite was synthesized in two steps (Figure 5A). Treatment of propanediol in dimethylformamide with sodium hydride followed by addition of hexadecyl bromide and catalytic potassium iodide gave 1-*O*-hexadecyl-1,3,-propanediol in a single step as previously observed;^45^ recrystallization with hexane yielded white crystals of excellent purity. The phosphoramidite was synthesized under standard conditions using 2-cyanoethyloxy(*N*,*N*-diisopropylamino)phosphonamidic chloride. The phosphoramidite was then conjugated to the 5′ end of LNA gapmers 33-N, 33-O, and 33-P via solid-phase synthesis (Figure 5A).

Besides direct recognition of cell-surface receptors by conjugated small molecules, conjugation approaches may change the biophysical properties of the oligonucleotide, which may affect their cell uptake in a more indirect way. For example, a variety of amphiphilic oligonucleotide conjugates have been shown to assemble into micelle-type structures. Previous work has described very long polymer conjugates that induce self-assembly of oligonucleotides into clusters,^46^ as well as oligonucleotides with shorter hydrophobic tails that assemble around a liposomal core.^47^

Therefore, we tested whether our conjugates self-assemble using dynamic light scattering (DLS). We found that conjugation of a hexadecyloxypropyl tail clearly induced assembly of oligonucleotides into clusters (Figure 5B). Our work demonstrates that even a simple hydrophobic conjugate may be sufficient to induce efficient self-assembly into clusters or micelles.

Clustering or assembly of oligonucleotides often correlates with enhanced uptake and activity, across multiple size scales and likely via multiple mechanisms.^48^, ^49^ Nevertheless, in our case, despite the efficient self-assembly observed for the lipid tail conjugates, the biostable hexadecyloxypropyl conjugates showed reduced silencing efficacy in cells (Figure 5C). A highly lipophilic tail might lead to stronger association with cell membranes but fail to release active oligonucleotide into the cytoplasm.^50^ We therefore generated biocleavable analogs based on a section of phosphodiester (PO)-linked DNA between the lipid tail and the PS gapmer ASO (Figure 5A). Nuclease cleavage at the PO linkages would allow release of the gapmer from the lipid tail inside endosomes (M.W. Lindholm et al., 2016, Oligonucleotide Therapeutics Society, conference). We found that treatment of cells with these biocleavable constructs produced similar potency silencing of *ADAM33* relative to the analogous unconjugated gapmers but did not provide a potency advantage in vitro (Figure 5C). However, hydrophobic conjugates of gapmer oligonucleotides may provide an advantage for tissue distribution or other in vivo properties, and our data point to the importance of making hydrophobic conjugates in a biocleavable manner, to avoid any membrane entrapment and associated potency reduction.

### ASOs Designed to Homologous Regions of the Mouse *Adam33* Transcript Are Also Active

To explore the biological role of *ADAM33*/*Adam33* in asthma progression, we have developed a series of mouse models.^7^ We therefore set out to identify analogs of the human lead ASOs that could silence mouse *Adam33* expression. The mouse and human *ADAM33*/*Adam33* transcripts are relatively well conserved in the coding region but diverge substantially toward the 3′ end of the open reading frame (ORF) and in their 3′ UTR sequences (for example, the mouse transcript has a much shorter UTR sequence than the human transcript).

**Figure 6:**
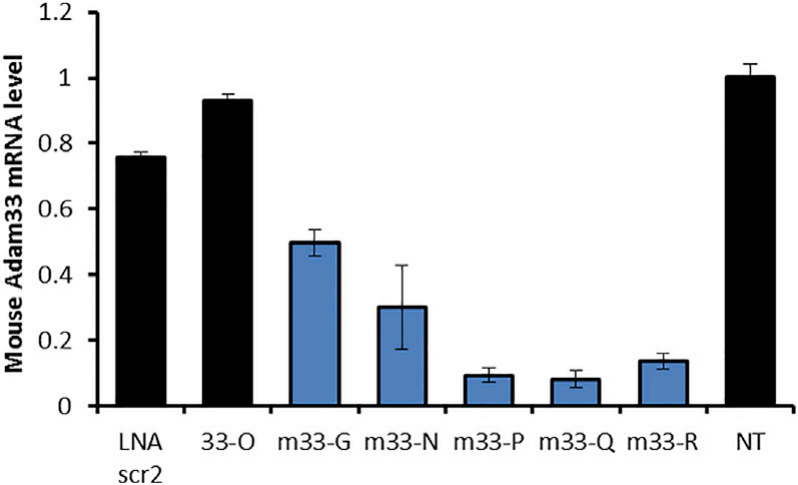
Identification of ASOs that Provide Highly Effective Silencing of Mouse *Adam33* Expression in Mouse Embryonic Fibroblasts Oligonucleotides were delivered at 50 nM with Lipofectamine RNAiMAX. Error bars represent the SD of biological replicates. Mouse-targeted sequences are shown in Table 1 and were made as fully phosphorothioate (PS) 3-9-3 LNA gapmers (the same chemical architecture as the human sequences). The sequence of LNA-scr2, also a PS 3-9-3 gapmer, is 5′-AACacgtctataCGC-3′. 33-O is the human sequence from Figure 3, included as an additional negative control.

Two of our active ASOs targeting the human *ADAM33* transcript also had significant sequence homology to the mouse transcript (G and N) (Table 1). Although the sequence conservation in the 3′ UTR was poor, we did an analysis of the conservation of structural features using T-Coffee^51^ and designed targets to regions of conserved structure (P and Q, near the end of the ORF, and R, in the 3′ UTR) (Table 1). These five sequences were synthesized as 3-9-3 LNA gapmers and tested for their ability to inhibit *Adam33* expression in mouse embryonic fibroblasts (Figure 6).

**Table 1:** Design of Mouse Analogs of Lead ASO Sequences

Small numbers refer to the base numbering within the target transcripts (in each case, human on top and mouse below). Gray highlighting in the mouse ASOs represents non-sequence-conserved residues relative to the human ASOs. While P, Q, and R showed only modest sequence conservation, they showed a greater degree of structural conservation as predicted by T-Coffee. RefSeq accession numbers of the sequences used for alignments were as follows: human, NM_025220.3 (3,573 nt total length, where the ORF ends at 2,569), and mouse, NM_033615.2 (3,165 nt total length, where the ORF ends at 2,694).

The most active ASOs in terms of silencing mouse *Adam33* expression were those targeting the 3′ end of the ORF or the 3′ UTR (Figure 6), which were also the three sequences that were less well conserved relative to the human hits. In future studies, we will test these sequences in mouse models to further explore the role of *ADAM33*/*Adam33* in asthma and the potential for oligonucleotides to play a therapeutic role in its treatment.

## Discussion

Single-stranded oligonucleotides are amenable to simple delivery approaches (including free uptake by simply delivering naked oligonucleotides into the blood or the CNS), which has led to a significant drive to develop single-stranded oligonucleotides, including fully^13^, ^14^ or partly^23^, ^24^ single-stranded oligomers that can engage the RNAi machinery (RISC). These are appropriate for gene silencing applications in which recruitment of RISC is required—for example, in the context of allele-selective silencing of mutant Huntingtin, RISC-dependent approaches showed far greater selectivity (>30-fold)^13^, ^52^, ^53^ compared with simpler steric blocker ASOs (∼3- to 6-fold).^54^, ^55^

However, for silencing nuclear-localized transcripts, recruitment of RISC may not be ideal. *ADAM33* mRNA is ∼90% nuclear localized even though it is a protein-coding transcript.^56^ RNase H is predominantly localized in the nucleus^57^ and plays a role in genome defense, including elimination of R loops.^58^ After gapmer-induced RNase H cleavage of a target RNA, the 3′ fragment is predominantly degraded by the nuclear enzyme 5′-3′ exoribonuclease 2 (XRN2).^59^ Nuclear RNAs including intronic sequences are vulnerable to cleavage induced by gapmer ASOs,^60^, ^61^, ^62^ and in the context of noncoding RNAs, it was observed that ASOs are less sensitive to subcellular localization of their targets, while siRNAs tend to be more effective at silencing ncRNAs that are predominantly cytoplasmic.^63^ This empirical tendency exists even though a cytoplasmic mechanism has also been described for gapmer ASO action^64^ and RNAi factors are present and active in human cell nuclei.^65^ Our current study suggests that it is important to consider subcellular localization of the target when selecting a preferred oligonucleotide class for silencing, even if the target is a protein-coding mRNA.

Our results should not be taken as a statement about the relative value or potency of ASO and siRNA silencing in general. For many targets, particularly in vitro, it is easier to find potent siRNAs than potent ASOs. The siRNA-Argonaute and ASO-RNase H approaches should be considered complementary rather than redundant.

In this project, multiple RNase H-dependent LNA gapmers were highly effective silencing agents for *ADAM33* mRNA, while siRNAs failed to yield potent silencing. Because gymnotically delivered oligonucleotides showed no toxicity and high efficiency in cultured cells, they are ideal for further *ADAM33* studies. We are exploring the delivery of anti-*Adam33* oligonucleotides to mouse airways and the therapeutic potential of ASO-mediated inhibition of *ADAM33* for reversing pathological airway remodeling in asthma and other chronic lung diseases, including chronic obstructive pulmonary disease (COPD)^66^, ^67^ and sarcoidosis.^68^

## Materials and Methods

### General Methods

^1^H-, ^13^C-, and ^31^P-NMR spectra were recorded on Bruker DPX or Bruker AV NMR spectrometers operating at 400, 101, and 162 MHz, respectively. Small molecules were analyzed using a Waters TQD mass spectrometer equipped with a triple quadrupole analyzer. Samples were introduced to the mass spectrometer via an Acquity UltraPerformance Convergence Chromatography (UPC^2^) system, including a UPC^2^ Waters HSS C18 SB column (100 mm × 3.0 mm × 1.8 μm) gradient 90% CO_2_:10% methanol modifier (25 mM ammonium acetate) to 60% CO_2_:40% methanol modifier (25 mM ammonium acetate) in 3 min at a flow rate of 1.5 mL/min. The makeup flow (methanol/1% formic acid) was pumped at a flow rate of 0.45 mL/min into the mass spectrometer. Mass spectra were recorded using positive ion electrospray ionization.

### Oligonucleotides

siRNAs were designed using the Whitehead siRNA selection server (http://sirna.wi.mit.edu). ASOs were manually designed using the predicted secondary structure of the mRNA (from mFold^69^), the predicted self-structure of the oligonucleotide, and the predicted specificity (from NCBI BLAST).

The first set of siRNAs for sequence screening was purchased from Integrated DNA Technologies (IDT). All other oligonucleotides were synthesized in-house at a 1-μmol scale on Applied Biosystems 394 DNA/RNA synthesizers with Unylinker (ChemGenes) or nucleoside-loaded CpG supports and standard detritylation and capping reagents. Activation was achieved with 5-benzylthio-1*H*-tetrazole (BTT, 0.3 M in acetonitrile). Oxidation was achieved using 0.02 M iodine in a mixture of tetrahydrofuran (THF), water, and pyridine. Sulfurization was accomplished with the 1,2,4-dithiazolines EDITH (Link Technologies) or DDTT (0.1 M, ChemGenes). RNA, 2′F-RNA, and 2′OMe-RNA phosphoramidites (ChemGenes) were dissolved to a concentration of 0.15 M in anhydrous acetonitrile immediately before use. LNA and MOE phosphoramidites were synthesized by standard methods^33^, ^70^, ^71^ from 3′-hydroxyl precursors (Rasayan) using 2-cyanoethyloxy(*N*,*N*-diisopropylamino)phosphonamidic chloride and were used at 0.1 or 0.15 M in acetonitrile, with the exception of LNA 5-MeC (LNA-C is actually LNA 5-Me-C), which was dissolved in a 3:1 mixture of THF:acetonitrile. Coupling times for all modified phosphoramidites were 10 min. Coupling yields for all nucleoside phosphoramidites were >98%. Coupling yields for the hexadecyloxypropyl phosphoramidite were good as long as the quality of the phosphoramidite was excellent. During one experiment, we used a batch of hexadecyloxypropyl phosphoramidite with somewhat lower purity and the coupling yield dropped to 50%.

Unmodified RNA was deprotected using a 3:1 ratio of NH_4_OH/EtOH for 48 hr at room temperature (RT). The RNA 2′OH tert-butyldimethylsilyl protecting group was removed with a 4:1 DMSO/triethylamine trihydrofluoride (TEA·3HF) solution at 65°C for 3 hr. The reaction was cooled to RT and then precipitated by the addition of 3 M NaOAc (25 μL) and BuOH (1 mL). The mixture was centrifuged at 4°C for 5 min at 8,000 rpm, washed with 70% EtOH, and air dried, and the pellet was resuspended in RNase-free water. 2′-modified RNA, LNA, and DNA were deprotected with concentrated NH_4_OH at 55°C overnight.

Oligonucleotides were evaporated to dryness by rotary evaporation and then resuspended in 1 mL RNase-free water. If liquid chromatography-mass spectrometry (LC-MS) and analytical PAGE (described later) indicated that the oligomer was sufficiently pure, it was desalted with a Nap-10 column (GE Healthcare) and used directly. All oligonucleotides were characterized on Bruker MicrOTOF Ultimate 3000 or Agilent Q-TOF LC-MS systems with electrospray ionization and time-of-flight analysis, using negative ionization mode. All sequences and mass spectrometric data are provided in Tables S1–S5.

20 μM working stocks of siRNAs were prepared by annealing the sense and antisense strands in a final 2.5× PBS buffer. The solutions were heated at 95°C for 10 min and then cooled to RT at a rate of 1°C per minute.

### Oligonucleotide Purification and Electrophoresis

Approximately 20 A_260_ units (preparative gel) or 0.1 A_260_ units (analytical gel) was loaded into a 20% polyacrylamide gel containing 7 M urea and run at 400 V for ∼3 hr. Analytical gels were visualized using Stains-All (Sigma). For preparative gels, the product band was briefly visualized by UV shadowing, excised from the gel, and incubated in RNase-free water overnight. The aqueous solution was then concentrated by rotary evaporator, resuspended in RNase-free water, and desalted via a Nap-25 column (GE Healthcare). The desalted oligonucleotide was evaporated to dryness again and resuspended in a small volume of RNase-free water.

Hexadecyloxypropyl-conjugated oligonucleotides were purified by 20% denaturing PAGE as earlier or by ion exchange chromatography using Agilent 1200 series high-performance liquid chromatography (HPLC), an Agilent PL-SAX column, and eluents containing 30% aqueous acetonitrile with increasing sodium perchlorate. Either method allowed us to remove all unconjugated oligonucleotide and obtain the pure conjugate.

### Cell Culture and Transfection

MRC-5 embryonic fibroblasts were maintained at 37°C and 5% CO_2_ in DMEM supplemented with 10% fetal bovine serum (FBS), 2% L-glutamine, 1% non-essential amino acid (NEAA), and 1% sodium pyruvate (all from Sigma). Cells were plated in 6-well plates at 150,000 cells/well (cationic lipid) or 25,000 cells/well (gymnotic) 24 hr before transfection, unless otherwise stated. Mouse embryonic fibroblasts (MEFs) were maintained in DMEM supplemented with 10% FBS and were plated in 6-well plates at 100,000 cells/well 24 hr before transfection.

Oligonucleotides were transfected at a 50 nM concentration for a single dose or decreasing doses for dose responses. Cells were transfected using RNAiMAX (Life Technologies) using 0.75 μL of lipid per 1 μL of oligonucleotide (siRNA) or 0.67 μL of lipid per 1 μL of oligonucleotide (LNA) in Opti-MEM (Life Technologies). Gymnotic delivery was achieved using a 1 or 3 μM oligonucleotide concentration in full cell culture media. Cells were harvested for RNA analysis 3 days after transfection (lipid) or 7 days after treatment (gymnotic) unless otherwise stated.

### RNA Harvest and Real-Time qPCR

Total RNA from cells was harvested 3 days post-transfection (lipid transfection) or 7 days post-transfection (gymnotic) unless otherwise stated. After washing each well with 1 mL of PBS, 1 mL of RiboZol (Amresco) was added to each well, incubated for 2 min at RT, and transferred to a 1.5-mL microcentrifuge tube. Chloroform (200 μL) was added to each tube, and the mixture was shaken vigorously for 1 min and then incubated at RT for 10 min. The mixture was centrifuged at 13,000 rpm for 20 min, and then the clear aqueous layer was transferred to a new 1.5-mL tube, avoiding any interphase. 2-propanol (600 μL) was added to the aqueous layer, followed by a 1-min vigorous shake and then a 20-min incubation at −20°C, and followed by a 15-min centrifugation at 14,000 rpm at 4°C. The resulting pellet was washed with ice-cold 70% ethanol, re-centrifuged at 8,000 rpm for 10 min at 4°C, and then briefly allowed to air dry. The pellet was resuspended in RNase-free water, heated to 55°C for 5 min, and then quantitated by UV spectroscopy.

1 μg of RNA was treated with 2 U of DNase I (Worthington Biochemical) for 10 min at 37°C, followed by 10 min at 75°C. RNA was reverse transcribed using a High-Capacity cDNA Reverse Transcription Kit (Life Technologies) per the manufacturer’s protocol.

qRT-PCR was performed using iTaq Supermix (Bio-Rad) on a Bio-Rad CFX96 real-time system. Data were normalized relative to levels of *GAPDH* mRNA. A primer and probe assay (IDT) specific for the *ADAM33* 3′ untranslated region was used (unless otherwise stated): forward primer, 5′-GGCCTCTGCAAACAAACATAATT-3′; reverse primer, 5′-GGGCTCAGGAACCACCTAGG-3′; probe, 5′-CTTCCTGTTTCTTCCCACCCTGTCTTCTCT-3′. A GAPDH primer-probe assay (IDT) was also used: forward primer, 5′-TGGTCCAGGGGTCTTACT-3′; reverse primer, 5′-CCTCAACGACCACTTTGT-3′; probe, 5′-CTCATTTCCTGGTATGACAACGAATTTGGC-3′. For mouse *Adam33* mRNA quantitation, we used TaqMan (Thermo Fisher Scientific) probe set Mm00459697_g1. All qRT-PCR experiments were performed in technical replicates. The qRT-PCR cycle was as follows: 95°C for 7 min (95°C for 15 s and 60°C for 30 s) × 40 cycles.

### DLS

The lyophilized gapmer ASO (33-O) and its hexadecyloxypropyl conjugate (33-O biostable conjugate) were dissolved in RNase-free water at a concentration of 20 nM and filtered with a 0.2-μm filter before measurement. The filtered samples were then transferred to a 384-well microplate and placed into a DynaPro PlateReader-II system (Wyatt Technology) for DLS measurement. The data were collected by averaging ten measurements (∼5 s each) for each sample.

### 2-Cyanoethyl (3-(Hexadecyloxy)Propyl) Diisopropylphosphoramidite

3-(hexadecyloxy)propan-1-ol was synthesized in one step according to the method of Yamano et al.^45^ and recrystallized from hexane. The crystalline product (200 mg, 0.67 mmol) was dissolved in dry CH_2_Cl_2_ and diisopropylethylamine (0.70 mL, 2.68 mmol, 4 equiv) was added while stirring at RT. 2-cyanoethyloxy(*N*,*N*-diisopropylamino) phosphonamidic chloride (0.18 mL, 0.8 mmol, 1.2 equiv) was added dropwise, and the solution was stirred for 45 min. After completion, 15 mL of CH_2_Cl_2_ were added and the organic phase was washed with saturated aqueous NaHCO_3_ and dried over MgSO_4_. The crude product was purified on a silica column with 50:50:1 Hex:EtOAc:NEt_3_ as eluent to afford the title compound as a colorless liquid (175 mg, 52% yield). R_f_ in EtOAc = 0.28. MS (ESI): found 501 (M+H); mass expected for (C_28_H_57_N_2_O_3_P + H = 501.4). ^1^H NMR (400 MHz, CDCl_3_) δ 0.89 (t, *J* = 6.85 Hz, 3H, CH_3_CH_2_) 1.19 (dd, *J* = 6.72, 3.42 Hz, 12H, 2 (CH_3_)_2_CHN) 1.26 (s, 26 H, 13 (CH_2_)_n_) 1.56 (quin, *J* = 6.94 Hz, 2H, OCH_2_CH_2_CH_2_) 1.88 (quin, *J* = 6.30 Hz, 2H, POCH_2_CH_2_CH_2_O) 2.64 (t, *J* = 6.60 Hz, 2H, CH_2_CN) 3.40 (t, *J* = 6.66 Hz, 2H, OCH_2_CH_2_CH_2_) 3.50 (t, *J* = 6.30 Hz, 2H, POCH_2_CH_2_CH_2_O) 3.54–3.65 (m, 2H, 2 CH) 3.65–3.79 (m, 2H, POCH_2_CH_2_CH_2_O) 3.79–3.93 ppm (m, 2H, POCH_2_CH_2_CN). ^13^C NMR (101 MHz, CDCl_3_) δ 14.1 (s, 1C, CH_3_CH_2_) 20.3 (d, *J* = 6.60 Hz, 1C, CH_2_CN) 22.7 (s, 1C, CH_2_CH_2_CH_3_) 24.5 and 24.63 (2 d, *J* = 7.70 Hz, 2x2C, (CH_3_)_2_CHN) 26.2 (s, 1C, OCH_2_CH_2_CH_2_) 29.3 (s, 1CH_2n_) 29.5 (s, 1CH_2n_) 29.6 (s, 2CH_2n_) 29.6 (s, 1CH_2n_) 29.7 (s, 5CH_2n_) 29.8 (s, 1CH_2n_) 31.5 (d, *J* = 7.34 Hz, 1C, POCH_2_CH_2_CH_2_O) 31.9 (s, 1CH_2n_) 43.0 (d, *J* = 11.74 Hz, 2C, 2 CH) 58.3 (d, *J* = 19.07 Hz, 1C, POCH_2_CH_2_CN) 60.7 (d, *J* = 17.61 Hz, 1C, POCH_2_CH_2_CH_2_O) 67.3 (s, 1C, POCH_2_CH_2_CH_2_O) 71.1 (s, 1C, OCH_2_CH_2_CH_2_), 117.6 ppm (s, 1C, CN). ^31^P NMR (162 MHz, CDCl_3_, ^1^H-decoupled) δ 147.56 ppm (s).

## Author Contributions

Conceptualization: H.M.H. and J.K.W.; Methodology: H.M.P., Y.N.T., H.M.H., and J.K.W.; Validation: H.M.P., Y.N.T., H.M.H., and J.K.W.; Investigation: H.M.P., P.M.K., and Y.Y.; Resources (Oligonucleotide and conjugate synthesis): H.M.P., P.M.K., A.J.D., M.P.M., L.N., and V.K.S.; Writing (draft): H.M.P. and J.K.W.; Review and Editing: all authors; Visualization: H.M.P. and J.K.W.; Supervision: H.M.H. and J.K.W.; Funding Acquisition: H.M.H. and J.K.W.

## Supplemental Information

Document S1. Figures S1 and S2, and Tables S1–S5

Document S2. Article plus Supplemental Information

## References

[1] Koppelman G.H., Sayers I. (2011). Evidence of a genetic contribution to lung function decline in asthma. J. Allergy Clin. Immunol..

[2] Tang M.L.K., Wilson J.W., Stewart A.G., Royce S.G. (2006). Airway remodelling in asthma: current understanding and implications for future therapies. Pharmacol. Ther..

[3] Global Asthma Network. (2014). The global asthma report. http://globalasthmareport.org.

[4] Van Eerdewegh P., Little R.D., Dupuis J., Del Mastro R.G., Falls K., Simon J., Torrey D., Pandit S., McKenny J., Braunschweiger K. (2002). Association of the ADAM33 gene with asthma and bronchial hyperresponsiveness. Nature.

[5] Lee J.H., Park H.S., Park S.W., Jang A.S., Uh S.T., Rhim T., Park C.S., Hong S.J., Holgate S.T., Holloway J.W., Shin H.D. (2004). ADAM33 polymorphism: association with bronchial hyper-responsiveness in Korean asthmatics. Clin. Exp. Allergy.

[6] Lee J.Y., Park S.W., Chang H.K., Kim H.Y., Rhim T., Lee J.H., Jang A.S., Koh E.S., Park C.S. (2006). A disintegrin and metalloproteinase 33 protein in patients with asthma: relevance to airflow limitation. Am. J. Respir. Crit. Care Med..

[7] Davies E.R., Kelly J.F., Howarth P.H., Wilson D.I., Holgate S.T., Davies D.E., Whitsett J.A., Haitchi H.M. (2016). Soluble ADAM33 initiates airway remodeling to promote susceptibility for allergic asthma in early life. JCI Insight.

[8] Puxeddu I., Pang Y.Y., Harvey A., Haitchi H.M., Nicholas B., Yoshisue H., Ribatti D., Clough G., Powell R.M., Murphy G. (2008). The soluble form of a disintegrin and metalloprotease 33 promotes angiogenesis: implications for airway remodeling in asthma. J. Allergy Clin. Immunol..

[9] Coussens L.M., Fingleton B., Matrisian L.M. (2002). Matrix metalloproteinase inhibitors and cancer: trials and tribulations. Science.

[10] Khvorova A., Watts J.K. (2017). The chemical evolution of oligonucleotide therapies of clinical utility. Nat. Biotechnol..

[11] Sharma V.K., Watts J.K. (2015). Oligonucleotide therapeutics: chemistry, delivery and clinical progress. Future Med. Chem..

[12] Deleavey G.F., Damha M.J. (2012). Designing chemically modified oligonucleotides for targeted gene silencing. Chem. Biol..

[13] Yu D., Pendergraff H., Liu J., Kordasiewicz H.B., Cleveland D.W., Swayze E.E., Lima W.F., Crooke S.T., Prakash T.P., Corey D.R. (2012). Single-stranded RNAs use RNAi to potently and allele-selectively inhibit mutant huntingtin expression. Cell.

[14] Lima W.F., Prakash T.P., Murray H.M., Kinberger G.A., Li W., Chappell A.E., Li C.S., Murray S.F., Gaus H., Seth P.P. (2012). Single-stranded siRNAs activate RNAi in animals. Cell.

[15] Wahlestedt C., Salmi P., Good L., Kela J., Johnsson T., Hökfelt T., Broberger C., Porreca F., Lai J., Ren K. (2000). Potent and nontoxic antisense oligonucleotides containing locked nucleic acids. Proc. Natl. Acad. Sci. USA.

[16] Kurreck J., Wyszko E., Gillen C., Erdmann V.A. (2002). Design of antisense oligonucleotides stabilized by locked nucleic acids. Nucleic Acids Res..

[17] Fluiter K., Mook O.R.F., Vreijling J., Langkjaer N., Højland T., Wengel J., Baas F. (2009). Filling the gap in LNA antisense oligo gapmers: the effects of unlocked nucleic acid (UNA) and 4′-C-hydroxymethyl-DNA modifications on RNase H recruitment and efficacy of an LNA gapmer. Mol. Biosyst..

[18] Stein C.A., Hansen J.B., Lai J., Wu S., Voskresenskiy A., Høg A., Worm J., Hedtjärn M., Souleimanian N., Miller P. (2010). Efficient gene silencing by delivery of locked nucleic acid antisense oligonucleotides, unassisted by transfection reagents. Nucleic Acids Res..

[19] Kini H.K., Walton S.P. (2007). In vitro binding of single-stranded RNA by human Dicer. FEBS Lett..

[20] Holen T., Amarzguioui M., Babaie E., Prydz H. (2003). Similar behaviour of single-strand and double-strand siRNAs suggests they act through a common RNAi pathway. Nucleic Acids Res..

[21] Pendergraff H.M., Debacker A.J., Watts J.K. (2016). Single-stranded silencing RNAs: hit rate and chemical modification. Nucleic Acid Ther..

[22] Allerson C.R., Sioufi N., Jarres R., Prakash T.P., Naik N., Berdeja A., Wanders L., Griffey R.H., Swayze E.E., Bhat B. (2005). Fully 2′-modified oligonucleotide duplexes with improved in vitro potency and stability compared to unmodified small interfering RNA. J. Med. Chem..

[23] Byrne M., Tzekov R., Wang Y., Rodgers A., Cardia J., Ford G., Holton K., Pandarinathan L., Lapierre J., Stanney W. (2013). Novel hydrophobically modified asymmetric RNAi compounds (sd-rxRNA) demonstrate robust efficacy in the eye. J. Ocul. Pharmacol. Ther..

[24] Alterman J.F., Hall L.M., Coles A.H., Hassler M.R., Didiot M.C., Chase K., Abraham J., Sottosanti E., Johnson E., Sapp E. (2015). Hydrophobically modified siRNAs silence huntingtin mRNA in primary neurons and mouse brain. Mol. Ther. Nucleic Acids.

[25] Nikan M., Osborn M.F., Coles A.H., Biscans A., Godinho B.M.D.C., Haraszti R.A., Sapp E., Echeverria D., DiFiglia M., Aronin N., Khvorova A. (2017). Synthesis and evaluation of parenchymal retention and efficacy of a metabolically stable O-phosphocholine-N-docosahexaenoyl-l-serine siRNA conjugate in mouse brain. Bioconjug. Chem..

[26] Fitzgerald K., White S., Borodovsky A., Bettencourt B.R., Strahs A., Clausen V., Wijngaard P., Horton J.D., Taubel J., Brooks A. (2017). A highly durable RNAi therapeutic inhibitor of PCSK9. N. Engl. J. Med..

[27] Monia B.P., Lesnik E.A., Gonzalez C., Lima W.F., McGee D., Guinosso C.J., Kawasaki A.M., Cook P.D., Freier S.M. (1993). Evaluation of 2′-modified oligonucleotides containing 2′-deoxy gaps as antisense inhibitors of gene expression. J. Biol. Chem..

[28] Inoue H., Hayase Y., Iwai S., Ohtsuka E. (1987). Sequence-dependent hydrolysis of RNA using modified oligonucleotide splints and RNase H. FEBS Lett..

[29] Agrawal S., Zhang X., Lu Z., Zhao H., Tamburin J.M., Yan J., Cai H., Diasio R.B., Habus I., Jiang Z. (1995). Absorption, tissue distribution and in vivo stability in rats of a hybrid antisense oligonucleotide following oral administration. Biochem. Pharmacol..

[30] Juliano R.L., Ming X., Carver K., Laing B. (2014). Cellular uptake and intracellular trafficking of oligonucleotides: implications for oligonucleotide pharmacology. Nucleic Acid Ther..

[31] Bennett C.F., Swayze E.E. (2010). RNA targeting therapeutics: molecular mechanisms of antisense oligonucleotides as a therapeutic platform. Annu. Rev. Pharmacol. Toxicol..

[32] Watts J.K., Corey D.R. (2012). Silencing disease genes in the laboratory and the clinic. J. Pathol..

[33] Martin P. (1995). A new access to 2′-*O*-alkylated ribonucleosides and properties of 2′-*O*-alkylated oligoribonucleotides. Helv. Chim. Acta.

[34] Singh S.K., Nielsen P., Koshkin A.A., Wengel J. (1998). LNA (locked nucleic acids): synthesis and high-affinity nucleic acid recognition. Chem. Commun. (Camb.).

[35] Obika S., Nanbu D., Hari Y., Andoh J.-i., Morio K.-i., Doi T., Imanishi T. (1998). Stability and structural features of the duplexes containing nucleoside analogues with a fixed N-type conformation, 2′-O,4′-C-methyleneribonucleosides. Tetrahedron Lett..

[36] Watts J.K. (2013). Locked nucleic acid: tighter is different. Chem. Commun. (Camb.).

[37] Souleimanian N., Deleavey G.F., Soifer H., Wang S., Tiemann K., Damha M.J., Stein C.A. (2012). Antisense 2′-deoxy, 2′-fluoroarabino nucleic acid (2′F-ANA) oligonucleotides: in vitro gymnotic silencers of gene expression whose potency is enhanced by fatty acids. Mol. Ther. Nucleic Acids.

[38] Soifer H.S., Koch T., Lai J., Hansen B., Hoeg A., Oerum H., Stein C.A., Kaufmann M., Klinger C. (2012). Silencing of gene expression by gymnotic delivery of antisense oligonucleotides.

[39] Winkler J. (2013). Oligonucleotide conjugates for therapeutic applications. Ther. Deliv..

[40] Wolfrum C., Shi S., Jayaprakash K.N., Jayaraman M., Wang G., Pandey R.K., Rajeev K.G., Nakayama T., Charrise K., Ndungo E.M. (2007). Mechanisms and optimization of in vivo delivery of lipophilic siRNAs. Nat. Biotechnol..

[41] Hostetler K.Y., Rybak R.J., Beadle J.R., Gardner M.F., Aldern K.A., Wright K.N., Kern E.R. (2001). In vitro and in vivo activity of 1-O-hexadecylpropanediol-3-phospho-ganciclovir and 1-O-hexadecylpropanediol-3-phospho-penciclovir in cytomegalovirus and herpes simplex virus infections. Antivir. Chem. Chemother..

[42] Prakash T.P., Graham M.J., Yu J., Carty R., Low A., Chappell A., Schmidt K., Zhao C., Aghajan M., Murray H.F. (2014). Targeted delivery of antisense oligonucleotides to hepatocytes using triantennary N-acetyl galactosamine improves potency 10-fold in mice. Nucleic Acids Res..

[43] Aldern K.A., Ciesla S.L., Winegarden K.L., Hostetler K.Y. (2003). Increased antiviral activity of 1-O-hexadecyloxypropyl-[2-(14)C]cidofovir in MRC-5 human lung fibroblasts is explained by unique cellular uptake and metabolism. Mol. Pharmacol..

[44] Hostetler K.Y. (2009). Alkoxyalkyl prodrugs of acyclic nucleoside phosphonates enhance oral antiviral activity and reduce toxicity: current state of the art. Antiviral Res..

[45] Yamano Y., Tsuboi K., Hozaki Y., Takahashi K., Jin X.-H., Ueda N., Wada A. (2012). Lipophilic amines as potent inhibitors of N-acylethanolamine-hydrolyzing acid amidase. Bioorg. Med. Chem..

[46] Edwardson T.G., Carneiro K.M., Serpell C.J., Sleiman H.F. (2014). An efficient and modular route to sequence-defined polymers appended to DNA. Angew. Chem. Int. Ed. Engl..

[47] Banga R.J., Chernyak N., Narayan S.P., Nguyen S.T., Mirkin C.A. (2014). Liposomal spherical nucleic acids. J. Am. Chem. Soc..

[48] Ezzat K., Aoki Y., Koo T., McClorey G., Benner L., Coenen-Stass A., O’Donovan L., Lehto T., Garcia-Guerra A., Nordin J. (2015). Self-assembly into nanoparticles is essential for receptor mediated uptake of therapeutic antisense oligonucleotides. Nano Lett..

[49] Subramanian R.R., Wysk M.A., Ogilvie K.M., Bhat A., Kuang B., Rockel T.D., Weber M., Uhlmann E., Krieg A.M. (2015). Enhancing antisense efficacy with multimers and multi-targeting oligonucleotides (MTOs) using cleavable linkers. Nucleic Acids Res..

[50] Nishina T., Numata J., Nishina K., Yoshida-Tanaka K., Nitta K., Piao W., Iwata R., Ito S., Kuwahara H., Wada T. (2015). Chimeric antisense oligonucleotide conjugated to α-tocopherol. Mol. Ther. Nucleic Acids.

[51] Notredame C., Higgins D.G., Heringa J. (2000). T-Coffee: a novel method for fast and accurate multiple sequence alignment. J. Mol. Biol..

[52] Hu J., Liu J., Corey D.R. (2010). Allele-selective inhibition of huntingtin expression by switching to an miRNA-like RNAi mechanism. Chem. Biol..

[53] Fiszer A., Mykowska A., Krzyzosiak W.J. (2011). Inhibition of mutant huntingtin expression by RNA duplex targeting expanded CAG repeats. Nucleic Acids Res..

[54] Gagnon K.T., Pendergraff H.M., Deleavey G.F., Swayze E.E., Potier P., Randolph J., Roesch E.B., Chattopadhyaya J., Damha M.J., Bennett C.F. (2010). Allele-selective inhibition of mutant huntingtin expression with antisense oligonucleotides targeting the expanded CAG repeat. Biochemistry.

[55] Hu J., Matsui M., Gagnon K.T., Schwartz J.C., Gabillet S., Arar K., Wu J., Bezprozvanny I., Corey D.R. (2009). Allele-specific silencing of mutant huntingtin and ataxin-3 genes by targeting expanded CAG repeats in mRNAs. Nat. Biotechnol..

[56] Powell R.M., Wicks J., Holloway J.W., Holgate S.T., Davies D.E. (2004). The splicing and fate of ADAM33 transcripts in primary human airways fibroblasts. Am. J. Respir. Cell Mol. Biol..

[57] Suzuki Y., Holmes J.B., Cerritelli S.M., Sakhuja K., Minczuk M., Holt I.J., Crouch R.J. (2010). An upstream open reading frame and the context of the two AUG codons affect the abundance of mitochondrial and nuclear RNase H1. Mol. Cell. Biol..

[58] Lima W.F., Murray H.M., Damle S.S., Hart C.E., Hung G., De Hoyos C.L., Liang X.H., Crooke S.T. (2016). Viable RNaseH1 knockout mice show RNaseH1 is essential for R loop processing, mitochondrial and liver function. Nucleic Acids Res..

[59] Hori S., Yamamoto T., Obika S. (2015). XRN2 is required for the degradation of target RNAs by RNase H1-dependent antisense oligonucleotides. Biochem. Biophys. Res. Commun..

[60] Kamola P.J., Kitson J.D., Turner G., Maratou K., Eriksson S., Panjwani A., Warnock L.C., Douillard Guilloux G.A., Moores K., Koppe E.L. (2015). In silico and in vitro evaluation of exonic and intronic off-target effects form a critical element of therapeutic ASO gapmer optimization. Nucleic Acids Res..

[61] Burel S.A., Hart C.E., Cauntay P., Hsiao J., Machemer T., Katz M., Watt A., Bui H.H., Younis H., Sabripour M. (2016). Hepatotoxicity of high affinity gapmer antisense oligonucleotides is mediated by RNase H1 dependent promiscuous reduction of very long pre-mRNA transcripts. Nucleic Acids Res..

[62] Kasuya T., Hori S., Watanabe A., Nakajima M., Gahara Y., Rokushima M., Yanagimoto T., Kugimiya A. (2016). Ribonuclease H1-dependent hepatotoxicity caused by locked nucleic acid-modified gapmer antisense oligonucleotides. Sci. Rep..

[63] Lennox K.A., Behlke M.A. (2016). Cellular localization of long non-coding RNAs affects silencing by RNAi more than by antisense oligonucleotides. Nucleic Acids Res..

[64] Castanotto D., Lin M., Kowolik C., Wang L., Ren X.-Q., Soifer H.S., Koch T., Hansen B.R., Oerum H., Armstrong B. (2015). A cytoplasmic pathway for gapmer antisense oligonucleotide-mediated gene silencing in mammalian cells. Nucleic Acids Res..

[65] Gagnon K.T., Li L., Chu Y., Janowski B.A., Corey D.R. (2014). RNAi factors are present and active in human cell nuclei. Cell Rep..

[66] Wang X., Li L., Xiao J., Jin C., Huang K., Kang X., Wu X., Lv F. (2009). Association of ADAM33 gene polymorphisms with COPD in a northeastern Chinese population. BMC Med. Genet..

[67] Figarska S.M., Vonk J.M., van Diemen C.C., Postma D.S., Boezen H.M. (2013). ADAM33 gene polymorphisms and mortality. A prospective cohort study. PLoS ONE.

[68] Shaffiq A., Haitchi H.M., Pang Y.Y., Alangari A.A., Jones M.G., Marshall B.G., Howarth P.H., Davies D.E., O’Reilly K.M.A. (2012). A disintegrin and metalloprotease (ADAM) 33 protein in patients with pulmonary sarcoidosis. Respirology.

[69] Zuker M. (2003). Mfold web server for nucleic acid folding and hybridization prediction. Nucleic Acids Res..

[70] Koshkin A.A., Singh S.K., Nielsen P., Rajwanshi V.K., Kumar R., Meldgaard M., Olsen C.E., Wengel J. (1998). LNA (locked nucleic acids): synthesis of the adenine, cytosine, guanine, 5-methylcytosine, thymine and uracil bicyclonucleoside monomers, oligomerisation, and unprecedented nucleic acid recognition. Tetrahedron.

[71] Chillemi R., Greco V., Nicoletti V.G., Sciuto S. (2013). Oligonucleotides conjugated to natural lipids: synthesis of phosphatidyl-anchored antisense oligonucleotides. Bioconjug. Chem..

