# Supplementary material for: Locked Nucleic Acid Gapmers and Conjugates Potently Silence ADAM33, an Asthma-Associated Metalloprotease with Nuclear-Localized mRNA
Source: Mol Ther Nucleic Acids. 2017 Jun 21;8:158–68. doi: 10.1016/j.omtn.2017.06.012 (PMC5498289; doi:10.1016/j.omtn.2017.06.012)
Supplement: Document S1. Figures S1 and S2, and Tables S1–S5 [file mmc1.pdf]

OMTN, Volume *8*

Supplemental Information

Locked Nucleic Acid Gapmers and Conjugates Potently Silence *ADAM33*, an Asthma-Associated Metalloprotease with Nuclear-Localized mRNA

Hannah M. Pendergraff, Pranathi Meda Krishnamurthy, Alexandre J. Debacker, Michael P. Moazami, Vivek K. Sharma, Liisa Niitsoo, Yong Yu, Yen Nee Tan, Hans Michael Haitchi, and Jonathan K. Watts

Locked Nucleic Acid gapmers and conjugates induce potent silencing of *ADAM33*, an asthma-associated metalloprotease with nuclear-localized mRNA

Hannah M. Pendergraff, Pranathi Meda Krishnamurthy, Alexandre J. Debacker, Michael P. Moazami, Vivek K. Sharma, Liisa Niitsoo, Yong Yu, Yen Nee Tan, Hans Michael Haitchi, Jonathan K. Watts

Supporting Information:

Supporting Figure S1: Comparison of unmodified and fully modified duplex siRNA

Supporting Figure S2: qRT-PCR time course results, gymnotic delivery

Supporting Tables S1–S5: Sequences and mass spectrometry data

A

siRNA duplex	Sequence
HMH-1 FM	5′ $G_sU_sACCUGGAACUG_sU_sA$<br>$A_sA_sC_sC_sU_sU_sC$ A $UGGACCUUGAC_sA_sU$–p 5′

(All other sequences given in main text)

B

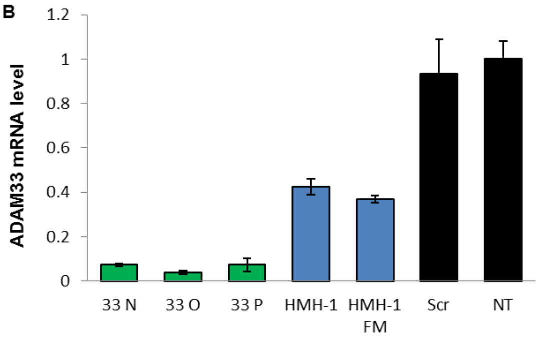

Figure S1. Silencing by a fully chemically modified duplex siRNA. **(A)** Sequence and modification pattern for the fully modified siRNA. Sequence modification codes are '$_s$' (phosphorothioate linkage); red: 2'F-RNA; blue: 2'OMe-RNA; p (5'-phosphate). Passenger strand is listed on top and guide strand underneath. **(B)** qPCR results showing ADAM33 level 3 days after transfection. Oligonucleotides were delivered with Lipofectamine RNAiMAX at 50 nM and normalized to a non-treated control. Error bars represent standard deviation of independent biological duplicates. Green bars are LNA gapmers, Blue bars are native or modified duplex siRNAs, Black bars are negative controls.

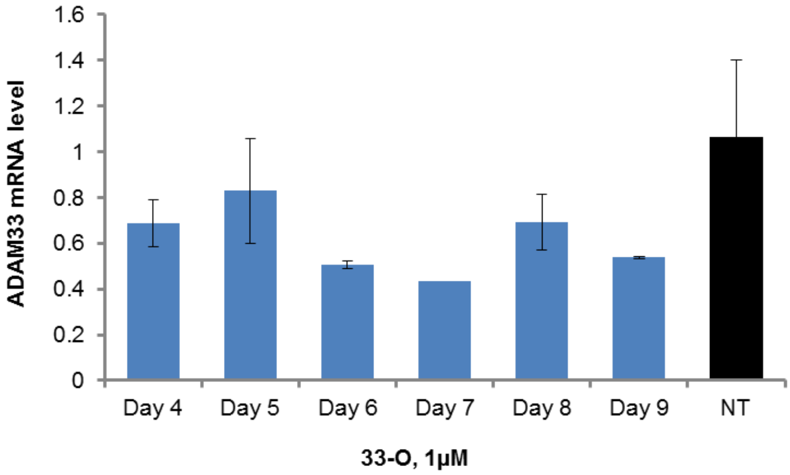

Figure S2. qRT-PCR time course results of gymnotic delivery at 1 μM concentration of ASO **33-O**. Oligonucleotides are delivered at 1μM dose and normalized to a non-treated control. Error bars are standard deviation of technical replicates. The degree of knockdown is intentionally chosen to be suboptimal (by using 1 μM rather than 3 μM ASO) to allow better discrimination of any differences in efficacy.

Table S1. siRNA sequences designed to target *ADAM33* mRNA, with mass spectrometry data. S represents sense strand, AS represents anti-sense strand.

siRNA	Sequence<br>Uppercase: RNA; Lowercase: DNA<br>For the bottom row only: **2'-F-RNA**; **2'-*O*-methyl-RNA;**<br>p: 5' phosphate; '$_s$' : phosphorothioate	Calculated mass (g/mol)	Found mass (g/mol)
**HMH-1**	5′ GGAAGUACCUGGAACUGUA<br>tt CCUUCAUGGACCUUGACAU 5′	S: 6124.7<br>AS: 6567.0	S: 6123.8<br>AS: 6565.8
**HMH-2**	5′ GGUGAGAGGUAGCUCCUAA<br>tt CCACUCUCCAUCGAGGAUU 5′	S: 6140.7<br>AS: 6566.0	S: 6139.8<br>AS: 6564.9
**HP--1**	5′ AGAAAGACAUGUUGGCUAU<br>tt UCUUUCUGUACAACCGAUA 5′	S: 6109.7<br>AS: 6552.0	S: 6108.7<br>AS: 6550.8
**HP- 2**	5′ GGGAGAUGCUCAUGGAAAC<br>tt CCCUCUACGAGUACCUUUG 5′	S: 6163.8<br>AS: 6543.0	S: 6162.7<br>AS: 6542.7
**HP-3**	5′ UGCUUGAGCUGGAGAAGAA<br>tt ACGAACUCGACCUCUUCUU 5′	S: 6164.8<br>AS: 6527.0	S: 6163.7<br>AS: 6525.7
**HP-4**	5′ UGGUGAACUCUGCGGGAGA<br>tt ACCACUUGAGACGCCCUCU 5′	S: 6156.7<br>AS: 6565.0	S: 6155.7<br>AS: 6564.8
**HP-5**	5′ CCCAACCACACGGAUCAUU tt<br>tt GGGUUGGUGUGCCUAGUAA 5′	S: 6572.1<br>AS: 6743.1	S: 6570.9<br>AS: 6742.9
**HP- 6**	5′ UGGCCCUGUGCAGGCUGAA tt<br>tt ACCGGGACACGUCCGACUU 5′	S: 6701.1<br>AS: 6644.1	S: 6701.0<br>AS: 6643.0
**HP- 7**	5′ AGUCCAGAUGCCAAGAUCC tt<br>tt UCAGGUCUACGGUUCUAGG 5′	S: 6652.1<br>AS: 6663.0	S: 6652.0<br>AS: 6662.9
**HP- 8**	5′ CCAGACGUUUACCUACUGG tt<br>tt GGUCUGCAAAUGGAUGACC 5′	S: 6606.0<br>AS: 6709.1	S: 6605.9<br>AS: 6708.0
**HP- 9**	5′ AGGGCGCCACAGUGGGCCU tt<br>tt UCCCGCGGUGUCACCCGGA 5′	S: 6739.1<br>AS: 6636.1	S: 6739.0<br>AS: 6636.0
**HP-10**	5′ GAUCAAGUCCAGAUGCCAA tt<br>tt CUAGUUCAGGUCUACGGUU 5′	S: 6676.1<br>AS: 6624.0	S: 6675.0<br>AS: 6623.8
**HP- 11**	5′ UAGCAACCAUAACUGCCAC tt<br>tt AUCGUUGGUAUUGACGGUG 5′	S: 6596.1<br>AS: 6704.0	S: 6594.9<br>AS: 6703.9
**Scr**	5′ AGUGGAGGGCGCCUGCCAC tt<br>tt UCACCUCCCGCGGACGGUG 5′	S: 6739.1<br>AS: 6636.1	S: 6739.05<br>AS: 6634.99
**HMH-1 FM**	5′ G$_s$U$_s$ACCUGGAACUG$_s$U$_s$A<br>A$_s$A$_s$C$_s$C$_s$U$_s$U$_s$C A UGGACCUUGAC$_s$A$_s$U-p 5′	S: 4977.3<br>AS: 7010.7	S: 4977.7<br>AS: 7009.8

Table S2. ss-siRNA sequences designed to target *ADAM33* mRNA, with mass spectrometry data.

**ss-siRNA**	**Sequence (5'-3')** ‘$_s$’ : phosphorothioate; **LNA**; **2’-F-RNA**; **2'-*O*-methyl-RNA**; **MOE**; P: 5’ phosphate	**Calculated mass (g/mol)**	**Found mass (g/mol)**
**ssi-HMH-1a**	**P-$U_sACAGUUCCAGG_sU_sA_sC_sU_sU_sC_sC_sA_sA$**	7131.0	7131.6
**ssi-HP-2**	**P-$G_sUUUCCAUGAGC_sA_sU_sC_sU_sC_sC_sC_sA_sA$**	7107.0	7106.6
**ssi-HP-3**	**P-$U_sUCUUCUCCAGC_sU_sC_sA_sA_sG_sC_sA_sA_sA$**	7091.0	7090.6
**ssi-HMH-1b**	**P-$U_sA_sCA_sGU_sUC_sCA_sGG_sUA_sC_sU_sU_sC_sC_sA_sA$**	7196.0	7195.7
**ssi-HMH-1c**	**P-$U_sA_sCA_sGU_sUC_sCA_sGG_sUA_sC_sU_sU_sC_sC_sA_sA$**	7108.1	7107.7
**ssi-HMH-1d**	**P-$U_sA_sCA_sGU_sUC_sCA_sGG_sUA_sC_sU_sU_sC_sC_sA_sA$**	7104.1	7103.6

Table S3. LNA gapmer ASOs designed to target h*ADAM33* mRNA, and LNA controls, with mass spectrometry data.

**LNA gapmer**	**Sequence (5'-3')** -‘$_s$’: phosphorothioate; LNA; Lowercase: DNA; HOP: Hexadecyloxypropyl	**Calculated mass (g/mol)**	**Found mass (g/mol)**
**33-G**	$T_sG_sA_s\ t_sc_sc_sg_st_sg_st_sg_sg_s\ T_sT_sG$	5023.0	5023.1
**33-H**	$A_sT_sG_s\ a_st_sc_sc_sg_st_sg_st_sg_s\ G_sT_sT$	5007.0	5007.1
**33-I**	$A_sA_sT_s\ g_sa_st_s\ c_sc_sg_s\ t_sg_st_s\ G_sG_sT$	5016.0	5016.1
**33-J**	$C_sA_sA_s\ t_sg_sa_s\ t_sc_sc_sg_st_sg_s\ T_sG_sG$	5015.0	5015.0
**33-L**	$T_sG_sT_s\ c_sa_st_sg_sg_st_s\ t_st_st_s\ C_sA_sG$	4996.0	4996.0
**33-M**	$G_sG_sT_s\ g_st_sc_sa_st_sg_sg_st_st_s\ T_sT_sC$	5012.0	5012.1
**33-N**	$A_sG_sG_s\ t_sg_st_sc_sa_st_sg_sg_st_s\ T_sT_sT$	5022.0	5022.0
**33-O**	$T_sC_sA_s\ t_st_st_st_sa_sg_sg_sa_sg_s\ C_sT_sA$	5003.0	5003.1
**33-P**	$T_sT_sC_s\ a_st_st_st_st_sa_sg_sg_sa_s\ G_sC_sT$	4994.0	4994.1
**33-Q**	$T_sG_sT_s\ t_sc_sa_st_st_st_sa_sg_s\ G_sA_sG$	5006.0	5006.0
**33-R**	$T_sC_sC_s\ g_st_sg_sg_sa_sa_sa_st_st_s\ G_sC_sA$	5027.0	5027.1
**lna ctrl**	$A_sT_sT_s\ t_st_sa_st_st_sc_sg_sg_sa_s\ G_sC_sT$	4980.0	4980.4
**lna scr2**	$A_sA_sC_s\ a_s{}^{m}c_sg_st_s{}^{m}c_st_sa_st_sa_s\ C_sG_sC$ (^{m}C is 5-methylcytosine)	4984.1	4983.5

Table S4. Biostable and biocleavable LNA gapmer ASOs designed to target h*ADAM33* mRNA, with mass spectrometry data.

**Conjugated LNA gapmer**	**Sequence (5'-3')**<br>'$_s$' : phosphorothioate; LNA; Lowercase: DNA;<br>HOP: Hexadecyloxypropyl	**Calculated mass (g/mol)**	**Found mass (g/mol)**
**33-N biostable**	$\mathrm{HOP_sA_sG_sG_s\ t_sg_st_sc_sa_sg_sg_st_s\ T_sT_sT}$	5400.2	5399.7
**33-O biostable**	$\mathrm{HOP_sT_sC_sA_s\ t_st_st_st_sa_sg_sg_sa_sg_sC_sT_sA}$	5364.2	5364.8
**33-P biostable**	$\mathrm{HOP_sT_sT_sC_s\ a_st_st_st_st_sa_sg_sg_sa_sG_sC_sT}$	5355.2	5355.8
**33-N biocleavable**	$\mathrm{HOP\ t\ t\ t\ t\ t\ t\ A_sG_sG_s\ t_sg_st_sc_sa_sg_sg_st_s\ T_sT_sT}$	7209.4	7208.8
**33-O biocleavable**	$\mathrm{HOP\ t\ t\ t\ t\ t\ t\ T_sC_sA_s\ t_st_st_st_sa_sg_sg_sa_sg_sC_sT_sA}$	7190.5	7189.3
**33-P biocleavable**	$\mathrm{HOP\ t\ t\ t\ t\ t\ t\ T_sT_sC_s\ a_st_st_st_st_sa_sg_sg_sa_sG_sC_sT}$	7181.5	7181.0

Table S5. LNA gapmer ASOs designed to target m*Adam33* mRNA, with mass spectrometry data.

**LNA gapmer**	**Sequence (5'-3')**<br>-'$_s$': phosphorothioate; LNA; Lowercase: DNA	**Calculated mass (g/mol)**	**Found mass (g/mol)**
**m33-G**	*(same as human **33-G** sequence, Table S3)*	5023.0	5022.1
**m33-N**	$\mathrm{A_sG_sG_sc_sa_st_sc_st_sc_sg_sg_st_sT_sT_sG}$	4991.9	4991.4
**m33-P**	$\mathrm{T_sA_sA_sg_sc_st_sc_sa_sg_sa_sg_st_sT_sC_sG}$	4999.0	4998.4
**m33-Q**	$\mathrm{G_sG_sT_sa_sa_sg_sc_st_sc_sa_sg_sa_sG_sT_sT}$	5025.0	5024.4
**m33-R**	$\mathrm{T_sC_sT_sa_st_sg_sa_sc_sa_sa_sc_sa_sG_sC_sT}$	4957.3	4956.5
